# The future is FAIR: a community framework for enhanced data management and data sharing

**DOI:** 10.1093/database/baag058

**Published:** 2026-09-25

**Authors:** Annarita Marrano, Carson Andorf, Jacqueline D Campbell, Ethalinda Cannon, Michael Coe, Laurel Cooper, Sarah Dyer, Peter W Harrison, Sunita Kumari, Sook Jung, Dorrie Main, John McNamara, David Molik, Sushma Naithani, Monica F Poelchau, Margaret E Staton, Peter Selby, Taner Z Sen, Marcela K Tello-Ruiz, Leonore Reiser

**Affiliations:** Phoenix Bioinformatics 39899 Balentine Drive, Suite 200, Newark, CA 94560, United States; Corn Insects and Crop Genetics Research Unit, USDA-ARS, Ames, IA 50011, United States; Corn Insects and Crop Genetics Research Unit, USDA-ARS, Ames, IA 50011, United States; Corn Insects and Crop Genetics Research Unit, USDA-ARS, Ames, IA 50011, United States; Cedar Lake Research Group LLC, Portland, OR 97293, United States; Department of Botany and Plant Pathology, Oregon State University, Corvallis, OR 97331, United States; European Molecular Biology Laboratory, European Bioinformatics Institute, Wellcome Genome Campus, Hinxton, Cambridge CB10 1SD, United Kingdom; European Molecular Biology Laboratory, European Bioinformatics Institute, Wellcome Genome Campus, Hinxton, Cambridge CB10 1SD, United Kingdom; Health Data Research UK, London NW1 2BE, United Kingdom; Cold Spring Harbor Laboratory 1 Bungtown Rd., NY 11724, United States; Department of Horticulture, Washington State University, Pullman, WA 99164, United States; Department of Horticulture, Washington State University, Pullman, WA 99164, United States; Department of Animal Sciences, Washington State University, Pullman, WA 99164, United States; Open Publishing Exchange, Kansas State University Libraries, Manhattan, KS 66506, United States; Department of Botany and Plant Pathology, Oregon State University, Corvallis, OR 97331, United States; Agricultural Research Service, National Agricultural Library, USDA, 10301 Baltimore Ave, Beltsville, MD 20705, United States; Department of Entomology and Plant Pathology, The University of Tennessee, Knoxville, Knoxville, TN 37996, United States; School of Integrative Plant Science, Plant Breeding and Genetics Section, Cornell University, Ithaca, NY 14850, United States; Crop Improvement and Genetics Research Unit, USDA-ARS, Albany, CA 94710, United States; Department of Bioengineering, University of California, 306 Stanley Hall, Berkeley, CA 94720, United States; Phoenix Bioinformatics 39899 Balentine Drive, Suite 200, Newark, CA 94560, United States; Cold Spring Harbor Laboratory 1 Bungtown Rd., NY 11724, United States; Phoenix Bioinformatics 39899 Balentine Drive, Suite 200, Newark, CA 94560, United States

## Abstract

The AgBioData consortium is a community-driven initiative supporting >40 genomic, genetic, and breeding (GGB) databases for agricultural research. Informed by the FAIR (Findable, Accessible, Interoperable, and Reusable) data principles, AgBioData aims to improve data management, sharing, and reuse across diverse biological data types. Through a National Science Foundation Research Coordination Network grant (award #2126334), the consortium engaged stakeholders via working groups, webinars, and community workshops to identify key data challenges and develop recommendations, tools, and training materials. Major outcomes include guidelines for standardizing genetic and genomic data, SNP harmonization protocols, recommendations for ontology usage, and an open-access curriculum for FAIR data literacy. Other key topics addressed in the first 3 years include data federation, improved single-cell data annotation, and advanced database interoperability. Surveys indicate an increase in community awareness and adoption of FAIR practices. AgBioData represents a scalable model for enhancing agricultural data infrastructure and sustainability, with ongoing efforts focused on AI readiness and the long-term support of curated data resources.

## Introduction

Over the last two decades, technological innovation has accelerated the generation and analysis of new data types, driving breakthrough discoveries in agricultural science. For instance, we can now assess the genome or transcriptome of individual cells and profile cell-to-cell variation at high resolution using single-cell sequencing technologies [1]. Additionally, we can quantify individual trait phenotypes using non-invasive digital imaging systems [2, 3]. However, while we are accelerating data discovery and analysis, we find it challenging to manage and share our data properly, and we continue to face numerous limitations when trying to reuse someone else’s data because it is not appropriately formatted or lacks crucial descriptors and metadata [4].

In 2016, Wilkinson *et al*. [5] published the Findable, Accessible, Interoperable, and Reusable (FAIR) principles to guide data generators, curators, and publishers on data stewardship and management, with the ultimate goal of maximizing the long-term care of digital assets. The core concept is that when data are properly shared, they can be easily found, interpreted, and, therefore, reused, increasing their potential value. FAIR data require the engagement of all key stakeholders in research, including scientists, publishers, and funding agencies, which demand detailed data management plans in grant proposals and that generated data to be shared in open-access repositories [6, 7]

At the same time, there has been a global shift towards open-access and standardized data sharing. Several initiatives involving database infrastructures have been established worldwide to define and promote best practices of data sharing and management. For example, the European infrastructure for life science data, ELIXIR (https://elixir-europe.org/), aims to facilitate data integration and reuse by bringing together Europe’s national research centres and core bioinformatics resources [8]. The Research Data Alliance (https://www.rd-alliance.org) was established as a community initiative to promote and facilitate the open sharing of research data across all scientific disciplines and the humanities. The National Microbiome Data Collaborative (NMDC, https://microbiomedata.org/) is a community-based initiative that seeks to address fundamental needs in microbiome data science by creating an open-source, integrated ecosystem and developing robust metadata curation processes based on community-driven standards [9].

Similarly to the NMDC, the AgBioData consortium (https://www.agbiodata.org/) applies a bottom-up participatory approach to break data-sharing barriers in agricultural research by embracing the FAIR principles and encouraging the community to adopt them. Established in 2015, AgBioData currently involves >40 genetic, genomic, and breeding (GGB) database resources (https://www.agbiodata.org/databases) and >360 members, including database curators, researchers, software developers, educators, and publishers [10]. To advance data capture, access, interoperability, and reusability in agricultural research, in 2021 AgBioData initiated a National Science Foundation Research Coordination Network (NSF RCN; Award Abstract # 2126334), with primary objectives to (a) engage the different stakeholders in the journey towards FAIR data through community workshops; (b) identify and address data priority issues by developing community-based standards; (c) educate current and future scientists to manage and share their data properly; and (d) advocate for long-term financial support of community databases that play a crucial role in making data more findable and reusable.

This manuscript provides an overview of the efforts and accomplishments achieved during the first 3 years (2021–24) of the AgBioData NSF RCN grant, their impact on increasing awareness and implementation of the FAIR principles within the community, and the consortium’s continued progress and future directions. Beyond documenting these activities, our primary objective is to derive a transferable framework and a set of actionable, generalizable lessons that can guide other research communities in developing FAIR data ecosystems within their respective domains. Accordingly, we focus on the processes and governance structures that enabled progress, including approaches for identifying community data priorities, establishing and sustaining working groups, fostering stakeholder engagement and participation, and assessing outcomes and impact. Rather than reiterating results that have already been reported in their dedicated publications, we highlight the organizational strategies, collaborative practices, and decision-making mechanisms that contributed to success and may serve as a model for similar community-driven initiatives.

## Community engagement and recruiting

**Figure 1:**
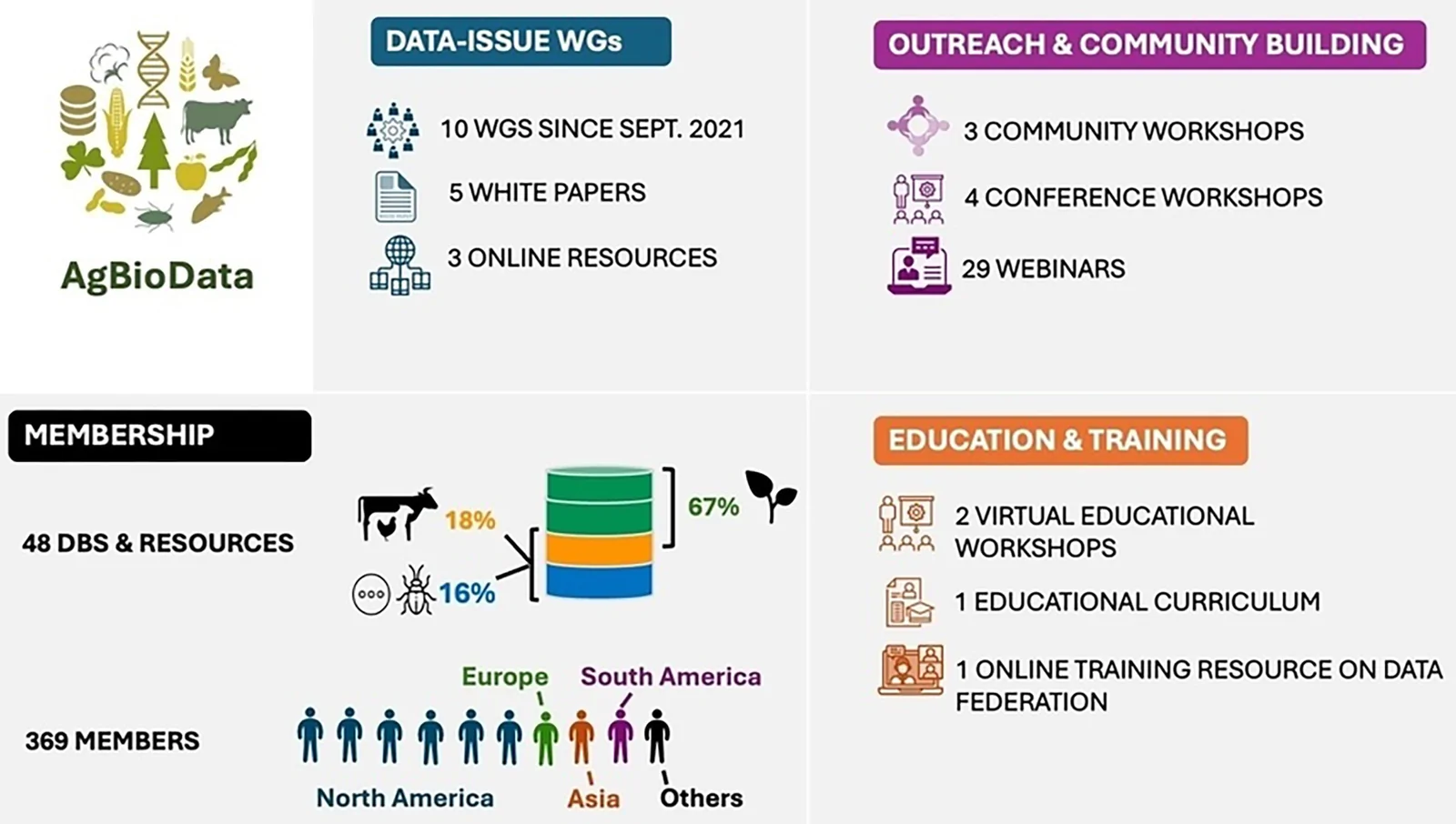
Summary of the activities, deliverables, and growth of the AgBioData consortium during the NSF RCN grant from September 2021–June 2025.

A core goal of the AgBioData NSF RCN grant was to assemble a broadly representative community actively engaged in identifying data priority issues and developing community-driven data standards. Since 2021, the AgBioData network has expanded its number of member databases and individual members, comprising researchers, database biocurators and developers, educators, group leaders, librarians, postdoctoral researchers, and students from 42 countries (Fig. 1).

We engaged with the community through (a) webinars, community workshops, and outreach at crucial conferences, and (b) actively recruiting a representative body of research scientists from various stakeholder communities.

Since September 2021, we have hosted >30 monthly webinars featuring experts on a range of topics (https://www.agbiodata.org/webinars). Monthly webinars fostered connections and professional networking within the AgBioData community, providing opportunities for education, training, and brainstorming on newly emerging data and technologies relevant to AgBioData members. For instance, discussions during monthly webinars led to highlighting the importance of phenotypic data standardization and management, the need for assessing existing single-cell expression (scRNA) data resources, and identifying the current gaps in scRNA metadata curation, data ingestion, and cell type annotations, and the use of artificial intelligence (AI) and natural language processing (NLP) for biocuration. These virtual roundtables proved to be an effective strategy for gathering opinions and perspectives from diverse stakeholders, understanding their needs, and brainstorming solutions to address them. Building on these virtual discussions, we identified critical issues and established new working groups, including the Phenotypic Data Standardization and Management (PDSM), the scRNA-Seq Biocuration, and the NLP for Biocuration Working Groups (WGs). Additionally, the availability of webinar recordings on our YouTube channel (https://www.youtube.com/@agbiodataconsortium748) makes the monthly webinars a valuable and enduring educational and training resource for our community.

Other opportunities to discuss data-related issues, brainstorm working group topics, and gather stakeholder feedback on works in progress were offered at the annual AgBioData community workshops. The workshops alternated between virtual and hybrid formats to enable maximum participation as well as the opportunity for in-person engagement. These workshops provided a forum for the WGs to present their progress and achievements, fostering cross-fertilization of ideas and identifying the consortium’s operational and strategic needs and future directions. The workshop format varied from year to year but always included presentation updates from the working groups, followed by breakout sessions in which participants addressed focused questions. Because many of our working groups have overlapping interests, this dedicated time for cross-fertilization ensured that working groups could benefit from the perspective of the broader community and coordinate related efforts. According to the post-workshop evaluation surveys, the meetings helped attendees better understand the work of the WGs and their positive impact on their research; most workshop participants acknowledged the role of these meetings in expanding their professional networks and initiating new collaborations.

Under the goal of building an inclusive community embracing the broad range of professional expertise related to agricultural research and education, we established the Outreach and Recruiting WG to recruit (a) stakeholders interested in joining the consortium and the AgBioData WGs, with a particular emphasis on early-career researchers and underrepresented communities, and (b) new GGB database resources. We successfully recruited 192 individual members and 6 new GGB resources, including biological database repositories (i.e. Ensembl [11] and the European Variation Archive [12]), community databases (i.e. iBeetle-Base [13], the Functional Annotation of ANimal Genomes—FAANG [14], and the Indian Crop Phenome Database, https://ibdc.dbtindia.gov.in/icpd/). The open-access journal microPublication, which partners with several AgBioData member databases to ensure data curation at the time of publication, joined as an affiliate (https://www.micropublication.org/). The Outreach and Recruiting working group also initiated relationships with programmes and organizations serving underrepresented communities, including the 1890 Land-Grant Institutions National Program and the Advancing Chicanos/Hispanics and Native Americans in Science (SACNAS) [15]. We invited them to present their missions, projects, and opportunities for connection and collaboration. Having a group specifically dedicated to outreach helped us to actively broaden our community, and we aim to continue these relationships and expand our efforts to engage with individuals and organizations that support FAIR data in agricultural research.

Monthly webinars, workshops, active recruitment, and participation in key conferences have proven effective in disseminating our work, growing our community by engaging influential figures in agricultural research, and raising awareness about the importance of FAIR data.

## Data issues working groups

The core activity of the AgBioData NSF RCN project was to identify and prioritize the most pressing data and metadata standardization needs, and to highlight possible solutions in collaboration with the community. We identified data priority issues by either surveying AgBioData members through online roundtable discussions or soliciting their input. Once identified, we established working groups (WGs) around the data-related issues, comprising volunteers with diverse professional and cultural backgrounds (Fig. 1). Since 2021, we have established over a dozen data-related working groups, focusing on various data types and challenges, including genetic variation, phenotypic data management, data reuse, single-cell data, and pan-genomes (Table S1). Each group had an average of 14 members, lasted ∼12 months, and delivered recommendations in the form of white papers, GitHub markdown pages, and web applications (Table S1; Fig. 1).

We survey the WG members annually to assess their experience within AgBioData WGs and improve WG organization and management. Most WG members report being satisfied with the WG organization and achievements. They agreed that participating in working groups helped them develop new skills and knowledge, and positively impacted their professional careers (e.g. authorship, new collaborations). In these evaluation surveys, the WG members also identified several challenges, including a lack of clear focus and goals, as well as conflicts between their professional responsibilities and volunteer commitments to the WGs. Building on this feedback, we developed a detailed WG framework outlining specific goals and the desired knowledge and skill sets for prospective members. By requesting relevant experience or expertise, we aim to recruit members who are genuinely engaged with the topic and motivated to make meaningful contributions. We also evaluated the progress and member engagement of each working group at 6- and 12-month intervals. Additionally, the programme coordinator observed WG meetings each quarter to ensure smooth operation and provide support when issues arise.

Over the years, we have observed that WGs tackling broad, complex data-related challenges often struggle with efficiency and productivity due to the diffuse nature of their objectives and the breadth of required expertise. To address this, we have refined our strategy by narrowing the scope of such WGs, particularly when dealing with multifaceted issues like phenotype data management. Instead of attempting to solve the entire problem, we established focused WGs dedicated to one or two well-defined components. This targeted approach has proven more effective in generating actionable outcomes and delivering meaningful, scalable improvements for our member databases.

The second major challenge frequently cited by WG members is the difficulty of balancing the responsibilities with their primary job commitments. To acknowledge their contributions and incentivize sustained engagement, we launched two initiatives in 2025: the AgBioData Champions Program (https://www.agbiodata.org/agb-championship) and the AgBioData Ambassador Program (https://www.agbiodata.org/agb-ambassador). The Champions Program recognizes WG members who demonstrate leadership, either by serving as chair or co-chair, or by taking the lead on significant deliverables such as drafting white papers or designing or developing software tools. To date, six Champions have been funded across five working groups. Champions have chaired working groups, spearheaded working group publications [16, 17], and led focused projects to advance working group deliverables. The Ambassador Program provides travel awards to WG members to support their participation in conferences, where they present AgBioData initiatives, WG progress, and key deliverables. Since 2025 we have supported 11 Ambassadors who presented posters or talks at conferences, including Plant and Animal Genomes, The International Society for Biocuration, American Society for Plant Biology, Advances in Genome Biology and Technology, Gordon Conference on Single Cell Approaches in Plant Biology, and the Global Bioinformatics Education Summit. These programmes are designed to honour the WG members’ efforts and commitment, sustain momentum within the working groups and the broader AgBioData community, spread the consortium’s deliverables to the larger agricultural research community, and expand the AgBioData network. In addition, we recognize the exemplary and sustained contributions of AgBioData members by bestowing awards for mission and leadership, best working group member, and other relevant efforts.

### Genomic and genetic data in agricultural research

The number of genetic, genomic, and transcriptomic datasets generated and published has increased significantly in recent decades [18]. These data are often used in genotype-to-phenotype association studies to understand the genetic control of traits of interest in agriculture, such as yield and stress tolerance. Despite the increased availability of genetic information, its reuse remains minimal [19]. The AgBioData Genotype-to-Phenotype Working Group has recently reviewed the factors that challenge the management and reuse of genetic and phenotypic data in plant science, including data heterogeneity and size, a lack of metadata and standardization, and inadequate database infrastructures for specific data types, notably high-throughput phenotypic data [20]. The development and adoption of standardized data collection and annotation protocols by data generators are crucial for ensuring data quality checks, archiving, and reuse for knowledge synthesis by biocurators and other researchers, ultimately facilitating connections between genotype and phenotype. The white paper also provides a comprehensive list of well-maintained public repositories and community databases for plant genomic, genotypic, and phenotypic data, including transcriptomic, proteomic, metabolomic data, and secondary knowledgebases. It also includes information on the metadata requirements and submission format used by these repositories to guide plant biologists in data sharing and incentivize data reuse.

Data reuse is the ultimate goal of open and FAIR science. Reusing existing data enables numerous new research applications and discoveries, while saving time and funding resources, and enhancing data value. Despite these appealing advantages, data reuse remains limited in many fields, including genomics [4], due to incorrect data formatting, missing metadata, or broken links to data repositories. The AgBioData Data Reuse (DR) Working Group addressed these challenges and provided recommendations in a white paper [19] for different stakeholders in agricultural research. In summary, the DR WG recommends (a) *data generators* submit data formatted according to community-based standards and ontologies and the complete metadata information as needed by specific data repositories; (b) *database managers* define a more comprehensive standardization of metadata across different databases, implement automatic formatting procedures during data submission, and make datasets citable (e.g. by assigning a DOI) to reward and reinforce FAIR data submission. The DR WG also generated recommendations for *publishers* and *funders*. For the former, they advise including data accessibility as a prerequisite for revision and publication. For the latter, they recommend checking for compliance with data management plans, financially supporting community databases, and investing in data literacy in science education (Table S1).

The release of and access to more genome assemblies has allowed many applications, notably the development of a species pan-genome, which is the sum of all the genome assemblies available for a species and their DNA variations (e.g. single nucleotide polymorphisms—SNPs; copy number variation—CNV, etc.) [21]. Recently, numerous pan-genomes have been published in agricultural research [22, 23]. Given the increasing need for guidelines to analyse, visualize, annotate, and share pan-genomic data, in 2021, we established the AgBioData Pan-Genome WG, which developed a comprehensive guide on what a pan-genome is and what it is used for, what software and pipelines are available for analysing, visualizing, and browsing pan-genome data, and how to archive and present them. This guide is available as a GitHub Markdown page and is freely accessible to anyone (Table S1).

SNPs are another popular data type widely used to assess a species’ genetic diversity and its impact on gene function and trait phenotypic variation. With advances in genotyping technologies and the release of improved genome assemblies, several SNP datasets for a single species can be complex to aggregate or compare due to discordant SNP nomenclature systems across databases or research projects. In this regard, the AgBioData Standards for Genetic Variation (SGV) WG recommends cross-linking genetic variant datasets to a reference genome of the International Nucleotide Sequence Database Collaboration (INSDC) and submitting variants to the European Variation Archive (EVA). The EVA assigns a reference single-nucleotide polymorphism identifier (rsID) to genetic variants at the same locus, facilitating variant mapping across genome assemblies and promoting interoperability among databases and genotyping platforms. The SGV WG has begun replacing temporary SNP identifiers with rsIDs in various crop databases and is coordinating with commercial genotyping service providers to utilize rsIDs in SNP arrays for agricultural species, as is widely done in human genetics.

Finally, advances in single-cell sequencing now offer unprecedented insights into the cellular and molecular bases of key agricultural phenotypes, opening new horizons for precision agriculture. To harness single-cell data analysis for robust genotype-to-phenotype prediction in agriculture, single-cell research communities must develop and adopt harmonized metadata schemas and standardized analytical workflows that support reproducible, cross‐study integration. In light of this need, there is an urgent imperative for researchers, funding agencies, and infrastructure providers to collaborate and develop resources on data standards, software pipelines, and shared repositories to support the analysis and application of single-cell data in agricultural genotype-to-phenotype research. The AgBioData scRNA-Seq Biocuration Working Group has recently developed a robust and sustainable network of researchers interested in applying single-cell genomics to elucidate the genotype-to-phenotype relationship in agricultural systems. The group shares members with the Plant Cell Atlas and Agricultural Genomes to Phenomes Initiative (Ag2PI) who have come together as a team to solve common problems. A comprehensive survey was also conducted to assess existing single-cell resources and identify the agricultural research community’s current needs. To hear from experts working on single-cell data from plants and animals, an in-person conference was held to discuss how to document and address the community’s needs (https://www.agbiodata.org/scrna-bio-workshop-agbt25).

### Data federation and interoperability

The lack of metadata and data standards, particularly for certain data types (e.g. high-throughput phenomics data), hinders data sharing among databases. A consortium-wide survey by the AgBioData Data Federation and Ontology Working Groups assessed the current status and future needs of member databases in data federation and ontology use [24]. The survey identified specific data types for which community standards and formats do not exist. It also recommended potential training on advanced data-sharing tools for database developers and managers, which led to the formation of the Data Federation Training WG in 2023. This working group (WG) developed training materials on the most commonly used data federation technologies within the agricultural, breeding, and scientific communities (Table S1), including Globus [25], SOLID (https://solidproject.org/), the Breeding API (BrAPI) [26], and GraphQL (https://graphql.org/). Each technology has a dedicated webpage containing a short description, a recorded presentation by an expert, a subjective cost estimate, example use cases, and an assessment of how the technology promotes FAIR data (https://github.com/AgBioData/DataFederation_WG/wiki). These training resources provide an easy way for the AgBioData community (or anyone interested in data federation and interoperability) to decide which data-sharing technology to implement and how to do it.

The Data Federation and Ontology WGs survey also sought feedback from the community about their awareness and use of ontologies. Since the advent of Gene Ontology (GO; [27]), there has been an incredible growth of ontology terms describing all biological facets (e.g. trait phenotypes) as they facilitate data findability and interoperability [28]. This massive growth, however, has not increased awareness of the importance of ontology use and its implementation [24]. Ontology use requires expert biocurator resources, funding to support them, and suitable tools to reduce the learning curve, which are often lacking or difficult to identify. To overcome these barriers to ontology use by member databases, the Ontology WG recommended exploring the several resources currently available on ontologies, such as those provided by the OBO Foundry (https://obofoundry.org/resources), the EBI-OLS (https://www.ebi.ac.uk/ols4), and Planteome (https://planteome.org/).

Using ontology terms aids data findability, access, interoperability, and reuse, as does the definition and adoption of a clear nomenclature system for genome assemblies and gene models [29]. While the rising number of new genome sequences and annotations released in the last decade has opened new fields of investigation, it has also introduced new data management issues, mostly related to the lack of a consistent and unambiguous genomic data nomenclature. It is often challenging to clearly and uniquely identify assemblies and unambiguously link gene model identifiers to their annotation datasets and source assemblies [30]. The AgBioData Genome Assembly and Annotation Nomenclature Working Group (GAAN WG) addressed this challenge and recommended a list of fields to be included when naming genome assemblies and annotations (Table S1). For instance, the *genome assembly identifier* should consist of (a) a species identifier, as generated by the Tree of Life project (https://id.tol.sanger.ac.uk); (b) a sample short name that uniquely identifies the material used to generate the genome (e.g. germplasm accession number, variety or landrace, or breed name); (c) an identifier of the consortium/project/group releasing the assembly; and (d) the assembly version. For a *gene model identifier*, the GAAN WG recommends including (a) the assembly prefix, (b) the annotation version, (c) the chromosome number (e.g. ‘1’ or ‘01’), (d) the entity type (e.g. ‘g’ for gene, ‘p’ for protein, ‘pan’ for pangene, and ‘t’ for transcript), and (e) an ID number, a unique identifier of minimum six numbers. The GAAN WG also generated a command-line tool to support and ease the adoption of the proposed nomenclature system. This Python-based tool, available at https://github.com/AgBioData/Genome-Assembly-and-Annotation-Nomenclature_WG, is a resource for systematically creating genome assembly and gene model identifiers.

## Educate and implement

Two crucial steps towards FAIR data in agricultural research are educating current and future scientists on better data management and providing a guide for navigating the database ecosystem when submitting their data or accessing publicly available data. The Education Working Group (EWG) and the FAIR Scientific Literature (FSL) WG focused on these tasks, involving educators, academic librarians, students, database curators, and publishers.

The EWG developed a modular curriculum for educators teaching undergraduate and graduate students the basics of biological databases and the FAIR principles [31]. The curriculum, named ‘AgBioData Curriculum for Ag FAIR Data’, includes seven modules focusing on different topics, including an introduction to biological databases, the FAIR principles, how to search, retrieve, and submit data, and how to use institutional libraries. Each module includes slides, recordings, and suggested activities, all of which are accessible at https://doi.org/10.5281/zenodo.13641594. This curriculum aims to increase scientists’ awareness of the importance of biological databases by supporting and teaching them how to manage their data correctly and navigate the database ecosystem. We envision the curriculum evolving by adding new modules on emerging data types and by initiating collaborations to translate the lessons into other languages (e.g. Spanish), so that a broad and diverse audience can interpret and utilize the curriculum. To support this work, we have established the Curriculum Implementation Awards (https://www.agbiodata.org/agb-education-implement-awards), which are small stipends for educators who wish to test, implement, or improve the curriculum. Through the Curriculum Implementation Awards, we aim to promote the adoption and maintenance of the AgBioData Curriculum for FAIR Ag Science and reinforce the education of current and future scientists on better data management. Additionally, we aim to collect feedback on the curriculum to facilitate further improvement.

The FAIR Scientific Literature (FSL) working group was established to identify bottlenecks in the publication-curation pipeline, identify existing or needed tools that can enhance the accuracy and throughput of literature curation and otherwise work towards making published agricultural data more FAIR. This group, comprising data-generating scientists and professionals from databases and journals, reviewed current literature, developed stakeholder personas, and identified barriers in making the scientific data FAIR particularly at the time of publication [(17)]. The publication of the ‘Nelson Memo’ in 2022, requiring that federally funded data be made available at the time of publication (6), presents a huge opportunity and challenge for databases. Making data not only open but FAIR requires coordination amongst data generators, publishers, and databases to ensure the published data and metadata are accurate and complete. However, for many researchers, technical limitations and the time commitment required for curation are barriers that limit the FAIRNess of published data. This could be addressed by the development of easy-to-use community tools to facilitate and simplify data curation, validation, and submission to facilitate data curation at the time of publication. Journals could partner with database curators to provide editorial review of data to ensure compliance—similar to the curator in the loop publishing model for microPublication Biology. Another barrier is that data generators and publishers do not always know where to deposit data, a problem with significant negative impacts on databases and curation workflows. To address this limitation, the FSL WG developed the AgBioDatabase Finder (https://www.agbiodata.org/databasefinder,(17)), a tool that helps identify the most suitable database for finding and submitting agricultural data based on the organism and types of data. The FSL WG also developed a list of recommended generalist repositories used by AgBioData databases (https://www.agbiodata.org/db-finder-repositories). A long-term goal of the working group is to address some of the structural and cultural barriers to ensure that published data are FAIR from the start.

## Tracking our progress

At its core, the AgBioData NSF RCN aims to facilitate communication to identify and prioritize the most pressing data and metadata standardization needs and collaborate on solutions to ensure increasing agricultural data FAIRness. We used surveys as a means to understand the impacts of the RCN activities on database professionals (people who work for member databases) and database stakeholders. These surveys included assessments of specific activities such as post-workshop surveys and the working group surveys described above, as well as a more comprehensive assessment to identify (a) potential shifts in stakeholder perception of data availability and understanding of data management, and (b) the perspective of database personnel on the impacts of AgBioData activities on data curation, interoperability, and exchange. We conducted baseline surveys of database users and database professionals separately in 2022, then conducted follow-up surveys with both groups in 2024. Participant samples from the two survey periods are not directly comparable for formal statistical estimation of change over time, but provide descriptive snapshots of those who responded at each point in time. Below, we highlight findings from the survey responses.

### Assessment of stakeholders’ use of GGB database resources and understanding of FAIR principles

We defined stakeholders as anyone using a GGB resource but not working for one. Eighty and 33 stakeholders participated in the survey in 2022 and 2024, respectively. Our respondents included academic researchers at all levels, breeders, bioinformaticians, and librarians. We inquired about their experiences with GGB databases, including searching, submitting, downloading, and reanalysing data, as well as their understanding of the FAIR principles (Table S2). We did observe some decrease in some aspects of GGB access and usage between 2022 and 2024 cohorts, which might partially be explained by differences in the stakeholder respondent communities. GGB stakeholders are often community-specific, our data collection did not enable us to correlate research community/DB and respondent answers. That is the pool of respondents was not the same, and may reflect community-level differences. Regarding the GGB user experience, both cohorts reported challenges with both data submission and retrieval/reanalysing, along with a need for more tutorials, FAQs, how to documents, and videos, or other resources for learning how to use the databases. Some solutions proposed in the working groups, such as streamlined submission processes and more universal and user-friendly tools, could address these issues. Still, these solutions require funding and time to be implemented.

Compared to the 2022 stakeholder sample, the 2024 survey respondents reported greater understanding of FAIR data principles and how to supervise the implementation of FAIR data management practices. In 2024, 72% of respondents ‘moderately’ or ‘strongly’ agreed that they were very familiar with the concept of FAIR data, compared to 52% of respondents in 2022; in 2024, 53% of respondents ‘moderately’ or ‘strongly’ agreed that they were very familiar with FAIR data management and could supervise these practices (questions Q1 and Q2 in Table S3). This increase is not necessarily a result of AgBioData initiatives but may reflect a general increase in awareness and understanding of FAIR data principles. The 2024 survey respondents also reported higher levels of FAIR implementation in the GGB databases than the 2022 sample. Specifically, a higher percentage of stakeholders agreed in 2024 that the GGB databases related to their work follow the FAIR principles during data submission (question Q5; Table S3), curate and catalogue data using standard terms (question Q6), and provide good guidelines on what metadata to submit when preparing and sharing data (question Q7). These results are encouraging and suggest that stakeholders are becoming more aware of how to properly format and share their data, as well as of the importance of doing so.

### Assessment of database professionals’ familiarity and implementation of FAIR data management guidelines

Database professionals (team members) include biocurators, software developers, research scientists, and anyone who manages and works for a GGB database resource. In 2022 and 2024, 25 and 38 database team members participated in the survey, respectively. Respondents reported high levels of familiarity and understanding of FAIR data management practices in 2022 and 2024 (Table S4). In 2024, 95% of participants ‘strongly’ or ‘moderately’ agreed that they are ‘very familiar with the concept of FAIR data principles and could explain them to others’, while 79% ‘strongly’ or ‘moderately’ agreed that they are ‘very familiar with technical/practical tools for FAIR data management and use them in my work’ and most of the remainder ‘slightly’ agreed with these statements. We also asked GGB database team members to rate their level of agreement or disagreement with statements about their experiences and opinions on the current status of FAIR data practices in GGB databases. Most respondents in both years agreed that the GGB databases they work for implement standard nomenclatures, metadata, common file formats, and user-friendly data system tools for submitting and downloading data, and provide guidelines for using the GGB database resource (Table S5). When 2024 respondents were asked to rate priorities for future development of GGB databases, 97% rated the application of community standards by publishers and the enforcement of data submission requirements as a ‘very important’ or ‘highest’ priority. Professional incentives from funders were similarly rated by 73% of respondents, along with the timely and up-to-date availability of curated data (84%). These priority areas coincide with some of the recommendations being developed by the FSL WG. One proposed solution is to develop strategic partnerships between databases and publishers/editors to ensure published data meet FAIR community standards. Another recommendation is to offer professional incentives in the form of ‘FAIR data’ badges for papers whose published data meet or exceed FAIR standards. One team member commented, ‘*Having good curated data continues to be a highly under-appreciated, under-funded activity. Raising awareness of the importance of manually curated data to leverage funded research projects and not to prevent the loss of important research results is also a very important task contributing to making data FAIR*.’ Some database team members also emphasized the importance of the long-term financial sustainability of GGB database resources to support high-quality, FAIR data in agricultural research, a core priority for the consortium.

### AgBioData’s impact on familiarity with FAIR data and the professional career of the survey participants

In the 2024 survey, we asked stakeholders (https://www.agbiodata.org/2024-survey-stakeh) and GGB database personnel (https://www.agbiodata.org/2024-survey-team) about their engagement with AgBioData and how this experience helped them become more familiar with and experienced in FAIR data management, as well as in their professional careers. All responding team members and 82% of stakeholders reported participating in at least one AgBioData event or activity, including conferences, workshops, working groups, the annual AgBioData community meeting, or webinars. When asked how this participation helped them personally or professionally, their comments emphasized the value of AgBioData for networking and establishing new collaborations, improving their knowledge of standards, software, and other technical issues, and recognizing the need for more funding to support AgBioData participation. For instance, a stakeholder reported that ‘*AgBioData has provided an incredible and collaborative environment for understanding the range of agricultural databases (e.g. how they support their communities with data storage and/or data dissemination). This understanding has allowed us to direct our efforts on designing solutions that help data producers, users, and databases incorporate FAIR data principles in their workflows. Professionally, through AgBioData, I met many people representing groups, and my group has started collaborations outside of AgBioData*.’ Similarly, a database curator who participated in the survey said, ‘*It is nice to see other curators out there having the same issues as me. The group helped start the discussion about what to do and provided some solutions*.’

Almost all the responding team members in 2024 (95%) reported that AgBioData helped them become more familiar with FAIR data management principles and practices. We specifically asked the GGB personnel if their database or related resources had implemented any recommendations, tutorials, or new technologies for data sharing and management due to their participation in AgBioData activities; 42% reported having ‘implemented changes that are (at least partly) due to their involvement in AgBioData activities.’ Another 42% indicated they plan to implement changes as recommended or learned from AgBioData. These responses confirm the effectiveness of the strategies used during the 3 grant years in building, educating, and supporting a community that moves together towards a FAIR data ecosystem in agricultural research.

## Lessons learned and future perspectives

In the first 3 years of our RCN project, we have made significant progress towards each of our aims, learned valuable lessons, and generated new ideas that inform our ongoing work. We doubled the number of participants, published white papers, delivered online resources for data generators and database personnel, and developed training materials on FAIR data management. Many of the deliverables include recommendations for infrastructure and tools that, if built, would significantly contribute to a more FAIR, sustainable, and AI-ready agricultural data ecosystem. Follow-on funding, in the form of new grants to support specific collaborative projects or centre-level activities, is needed to affect a transformation. More generally, our experience offers a transferable template that other research communities can adapt: Identify data priorities through participatory community roundtables, address them through time-boxed, narrowly scoped working groups, formally recognize and incentivize contributors, and evaluate impact through repeated stakeholder surveys.

Much of the success of this research coordination network can be attributed to having a dedicated programme coordinator who serves as both community and project manager. The programme coordinator bears considerable responsibility for the success and accomplishments of the consortium. The programme coordinator ensures fluid communication within and between the working groups and the AgBioData steering committee, tracks grant milestones, and provides extensive support in planning and organizing outreach initiatives. While the working group and steering committee members are volunteers who need to reconcile their commitments with work responsibilities, the programme coordinator is fully dedicated and motivated to the project’s fulfilment. We highlight this as a transferable organizational lesson: community initiatives of comparable scope should budget for dedicated coordination capacity rather than relying solely on volunteer effort. In our experience, a professional coordinator who complements rather than replaces a volunteer-driven structure is a decisive factor in sustaining momentum, maintaining cross-group communication and delivering on milestones, and we recommend that similar consortia plan for this role from the outset.

Over the years, we have learned that a defined focus with clear and achievable goals is crucial for a working group’s success, along with motivated leadership and a flexible meeting schedule. Also, specific data-related issues have multiple facets that cannot be adequately addressed in a 1-year working group (e.g. phenotypic data management). Prioritizing these multiple facets based on the needs of the consortium member databases and participants helped simplify the data-related issues and define a clear working group focus. Taking these lessons into account, we will continue to establish working groups as a primary strategy to address newly emerging data-related issues. For instance, in March 2025, we launched the Natural Language Processing (NLP) for Biocuration working group (https://www.agbiodata.org/nlp4biocuration) to explore existing NLP models and tools, their potential to speed up biocuration and coding, and their limitations. We will also explore other AI applications for data quality and management, and form new working groups to address the identified issues and needs. In addition, we have established the AgBioData Champions Program and the AgBioData Ambassador Program to recognize the efforts and time commitments of working groups’ members and incentivize their participation.

The long-term stability and sustainability of GGB databases and secondary knowledgebases that curate gene expression data, gene-gene networks, and pathways will be a core focus in the upcoming years of the consortium. Maintaining an open and FAIR agricultural data ecosystem is crucial, as public databases and other resources are the backbone of modern agricultural research, enabling groundbreaking discoveries. Still, most database resources rely on fragmented short-term public funding, repeatedly facing the threat of losing financial support, which can jeopardize their ability to serve the agricultural research community effectively and equitably. The escalating demands of data-intensive science and AI-ready datasets add further complexity to this challenge. Biodata resource sustainability is a global challenge [32], and it requires a cooperative framework to address these issues. The AgBioData Sustainability working group (https://www.agbiodata.org/working_groups/sustainability) will continue working on identifying and addressing common sustainability challenges affecting GGBs, taking into account the results of the sustainability survey (https://www.agbiodata.org/sust-survey), potential sustainability models (https://www.agbiodata.org/agbiodata_sustainability_models), recommendations, and the feedback gathered from our community. We will also quantify the utilization, scientific impact, economic impact, and projected financial requirements of AgBioData member databases. In line with a sustainable database ecosystem, we will also evaluate data federation schemes for the consortium, such as sharing personnel and software resources across the member databases.

In conclusion, with support from the National Science Foundation, the AgBioData Consortium has evolved into a mature and still expanding collaboration of database professionals, data curation specialists, research stakeholders, and data publishers. We have had success in achieving many of our deliverables and learned some valuable organizational lessons that can be applied to other similarly structured community projects. As we look forward to the next 10 years, we are exploring different avenues to ensure the sustainability of the Consortium as a whole, perhaps by establishing a non-profit entity. We plan to continue to build upon this community foundation to advance FAIR data in agricultural research by disseminating our deliverables, educating researchers and stakeholders on FAIR data management, and advocating for the implementation of recommended actions to enhance the adoption of FAIR principles and practices. We will continue to explore new data-related issues and evaluate how best to implement AI for data curation and/or supporting expert biocuration. Data standardization and biocuration are crucial for democratizing data and knowledge, now more than ever, in the current AI revolution [33]

## Supplementary Material

baag058_Supplemental_Files

## Data Availability

All data is provided in the text, supplementary files, or accessible through DOI links and URLS included in the text and online supplementary tables.
