# Supplementary material for: The future is FAIR: a community framework for enhanced data management and data sharing
Source: Database (Oxford). 2026 Sep 25;2026:baag058. doi: 10.1093/database/baag058 (PMC13613210; doi:10.1093/database/baag058)
Supplement: Supplementary file 1 [file baag058_supplemental_files.zip › Table_S1_v2 _07142026.pdf]

Table S1. Recommendations and deliverables of the AgBioData Issue working groups during the NSF RCN grant.

Working group (WG)	Current Status	Main focus	Recommendations	Deliverables
Data Federation (DF)	Ended	Identify barriers and best practices of data sharing among the AgBioData member databases	This WG identified that data sharing among databases is limited by<br><br>- Funding, time, and resources<br>- Lack of technical knowledge of sharing techniques<br>- Lack of community standards for some data types (e.g., phenotypic data)<br>- Incomplete metadata sharing<br>- Lack of users/stakeholders' awareness of the benefits of data sharing.<br><br>They recommend:<br>- developing training material on sharing techniques and data federation<br>- prioritizing improvements for specific phenotypic data types and formats<br>- studying how well metadata is shared<br>- increasing stakeholders' awareness of the benefits of data sharing.	Manuscript "**Data sharing and ontology use among agricultural genetics, genomics, and breeding databases and resources of the Agbiodata Consortium**," (Clarke et al. 2023; https://doi.org/10.1093/database/baad076).
Data Federation Training (DTF)	Ended	Develop training material on different solutions for data sharing and data federation	Database developers and managers can use the beginner's guide ondata-sharing technologies developed by the DTF WG. This guide includes short description of each technology, along with a recorded presentation by an expert, a subjective cost estimate, pros, cons, example use cases, and an assessment of how the technology promotes FAIR data..	A GitHub page with descriptions and training materials on the most used data-sharing techniques within the agricultural, breeding, and scientific community (https://github.com/AgBioData/DataFederation_WG/wiki).
Data Reuse (DR)	Ended	Identify and address challenges and limitations to data reuse in agricultural research.	The DR WG recommends the following to the different stakeholders to improve data reuse:<br><br>**DATA GENERATORS**<br><br>- Submit the data to the appropriate data repositories<br>- Submit complete meta-information, including recorded factors that were not relevant to the original study<br>- Adopt community-based data format and ontologies, if available<br>- Make all code used in the computational analysis available alongside raw and/or processed datasets<br>- To guard against data obsolescence, researchers need to incorporate thorough analysis workflows to enrich metadata for methodological detail<br>- Engage with Indigenous communities, practice responsible data stewardship, and use Indigenous ethics to determine data access<br><br>**DATABASES**<br><br>- Define a more comprehensive standardization of metadata across different databases, both in what is captured and how it is captured.<br>- Facilitate data submission by implementing automatic procedures to complete certain fields.<br>- Reward data submission, making the dataset(s) citable (e.g., assigning a Digital Object Identifier - DOI) or awarding the most reused datasets<br>- Provide more user-friendly tools that facilitate interconversion between formats without accompanying loss of metadata.<br><br>**PUBLISHERS**<br><br>- Provide more detailed templates that require researchers to fill in accessions or URLs and to include data accessibility as a criterion for reviewers to assess. Provide the possibility to link a GitHub repository with the analysis code, Open Science Framework material, or specific datasets to the submission<br><br>**FUNDERS**<br><br>- Checking for compliance with data management plans (DMPs)<br>- Financially support public infrastructure or provide more sustainable funding for biodata resources<br>- Invest in data literacy early in science education	- A GitHub page with compiled recommendations for data generators (https://github.com/AgBioData/Dhttps://doi.org/10.20944/preprints202401.0780.v1ata-Reuse?tab=readme-ov-file#1-accessing-the-dataset)<br><br>- The manuscript "***Data reuse in agricultural genomics research: challenges and recommendations***" by Hafner *et al.* (2025) (https://doi.org/10.1093/gigascience/giae106)

Table S1. Recommendations and deliverables of the AgBioData Issue working groups during the NSF RCN grant.

Working group (WG)	Current Status	Main focus	Recommendations	Deliverables
Education	Ended	Produce a high-level guide for educating students about biological digital resources and FAIR data management	Educating scholars about databases, how to use them, and the importance of making data FAIR would help current and future research students and professionals navigate the database ecosystem and encourage them to follow the FAIR guidelines.	- The AgBioData Curriculum for FAIR Ag Science (https://zenodo.org/records/14278084)<br><br>- Manuscript "**A teaching and training framework to promote findable, accessible, interoperable, and reusable data generation in agriculture**" (Marrano et al. 2025; https://doi.org/10.1093/database/baaf034)
FAIR Scientific Literature (FSL)	Active	- Identify bottlenecks in the publication-curation pipeline<br>- Identify sets of existing or desired biocuration resources to increase literature curation throughput and curation<br>- Publish recommendations and a roadmap for authors and publishers to increase the FAIRness of published research	Need for a tool to help researchers, reviewers and publishers to identify appropriate repositories for GGB data submission.	-Manuscript "**AgBioDatabase Finder: an online tool to help researchers find and submit agricultural genomic, genetic, and breeding data"** (Cabugos, et al. 2025; https://doi.org/10.17912/micropub.biology.001896)<br><br>-Database Finder Tool (https://www.agbiodata.org/databasefinder)
Genome Assembly and Annotation Nomenclature (GAAN)	Ended	Understand current challenges and potential solutions toward a standard gene ID nomenclature system.	A **genome assembly identifier** should include the following elements to be both human and machine-readable:<br><br>- Species identifier, as generated by the Tree of Life project<br>- Sample short name (e.g., accession/variety/landrace/breed/field location/colony/etc.)<br>- Consortium/Project/Group assembling<br>- Assembly version<br><br>A **gene model identifier** should include the following elements:<br><br>- Assembly prefix<br>- Annotation version<br>- Chromosome<br>- Entity type (e.g., g for gene, p for protein, pan for pan-gene, and t for transcript)<br>- ID number - a unique numeric identifier can be generated for each gene model within the genome<br>- Sub-genome and chromosome (optional)<br>- Haplotype assemblies (optional)<br>- Transcript isoforms (optional)	- Manuscript "**Guidelines for gene and genome assembly nomenclature**" (Cannon et al. 2025; https://doi.org/10.1093/genetics/iyaf006)<br><br>- The "AgBioData Assembly Name Generator," a Python-based tool to generate genome assembly and gene model identifiers (available at https://github.com/AgBioData/Genome-Assembly-and-Annotation-Nomenclature_WG)
Genotype-to-Phenotype (G2P)	Ended	Identify the limitation to proper data sharing of data types generated and used to cover the gap genotype-phenotype in plant science	- Database resources should implement standardized data models and emerging software components to facilitate genotypic and phenotypic data interoperability.<br><br>- Researchers should use automated management of data flows and implement data quality checks (QC) before sharing their data.<br><br>- Funders and database users should financially support existing community databases to enforce biocuration, data security and recovery, and data compliance with data-sharing policies.	Manuscript "**Genotype and phenotype data standardization, utilization and integration in the big data era for agricultural sciences**" (Deng et al. 2023 ; https://doi.org/10.1093/database/baad088)
Ontology	Ended	Identify and address the gaps and barriers to ontology use	This WG identified what limits ontology use from the AgBioData member database:<br><br>- Lack of expert biocurator resources<br>- Funding to support biocurators<br>- Suitable tools to lower the learning activation barrier<br>- An overall understanding of the importance of ontology use	Manuscript "**Data sharing and ontology use among agricultural genetics, genomics, and breeding databases and resources of the Agbiodata Consortium**" (Clarke et al. 2023 ;https://doi.org/10.1093/database/baad076)
Pan-genome	Ended	Provide an extensive description of pan-genomes and review current methods, pipelines, and tools for the construction and visualization of pan-genomes	Databases should provide the ability to download all or a subset of the pan-genes, with or without associated data as a table.	A GitHub markdown page with the recommended visualization and analysis tools of pan-genome data (https://github.com/AgBioData/Pan-genomes/blob/main/Pan-genome_resource.md)
Phenotypic Data Standardization and Management (PDSM)	Active	Address the need for standards, guidelines, and expertise for phenotypic data management that can be implemented by the consortium member databases and the large community of data users and generators.	-Need to build FAIR phenomic databases<br>-Adopt and enforce community data stanadards (like MIAPPE) and ontologies .<br>-Invest in large scale integrated phenomics studies<br>-Establish sustainable public funding model w possible private partners<br>-Support complete open and FAIR data policy implementation<br>-Strengthen coordination across agencies<br>-Invest in workforce education about FAIR data practies and phenomics data utilization	Manuscript "**The need for robust, FAIR phenomic databases supporting agricultural efficiency and resiliency**, by Callwood, J.,et al.2025 (https://doi.org/10.1093/scipol/scaf039)

**Table S1. Recommendations and deliverables of the AgBioData Issue working groups during the NSF RCN grant.**				
**Working group (WG)**	**Current Status**	**Main focus**	**Recommendations**	**Deliverables**
Single-cell RNA-Seq Biocuration	Active	- Create a cohesive community that will address the challenges related to scRNAseq metadata in plants and animals<br>- Establish a set of recommendations for member databases on the management of sc datasets<br>- Provide resources, tools, and standardized scRNAseq datasets for FAIR data meta-analysis.	In progress	Co-hosted an in-person workshop with the AG2PI Initiative to advance agricultural single-cell genomics (https://www.agbiodata.org/scrna-bio-workshop-agbt25)
Standards for Genetic Variation (SGV)	Active	Improve availability and reusability of genetic variation datasets and related metadata	- Genetic variant datasets should be deposited and cross-linked to an INSDC reference genome.<br><br>- Genetic variants should have an rsID to facilitate variant mapping on a new genome assembly and promote interoperability across databases and genotyping platforms.	- Replaced temporary SNP identifiers with rsIDs in various crop databases (e.g., 40K SNPs in SorghumBase) and mapped rsIDs to other cultivars (pan-genome sites)<br><br>- Coordinated with commercial providers of genotyping services to use rsIDs in Ag SNP arrays<br><br>-Manuscript: **Adoption of Standard Reference SNP Identifiers in Agricultural Genomics for Interoperability and Data Reuse** (Telo-Ruiz, et al. 2026; https://doi.org/10.1038/s41597-026-07208-0)
